# Effective educator–student relationships in nursing education to strengthen nursing students’ resilience

**DOI:** 10.4102/curationis.v39i1.1595

**Published:** 2016-06-10

**Authors:** Kathleen Froneman, Emmerentia du Plessis, Magdalena P. Koen

**Affiliations:** 1 School of Nursing Science, North-West University, Potchefstroom Campus, South Africa; 2 School of Nursing Science, North-West University, Mafikeng Campus, South Africa

## Abstract

**Background:**

Little research has been conducted in private nursing schools with regard to the educator–student relationship to strengthen the resilience of nursing students and to improve the educator–student relationship. An effective educator–student relationship is a key factor to ensure a positive learning climate where learning can take place and resilience can be strengthened.

**Purpose:**

The purpose was to explore and describe nursing students’ view on the basic elements required for an effective educator–student relationship to strengthen their resilience and the educator–student relationship.

**Method:**

This study followed an explorative, descriptive and contextual qualitative design in a private nursing education institution in the North West Province. Purposive sampling was used. The sample consisted of 40 enrolled nursing auxiliary students. The World Café Method was used to collect data, which were analysed by means of content analysis.

**Results:**

The following five main themes were identified and included: (1) teaching–learning environment, (2) educator–student interaction, (3) educator qualities, (4) staying resilient and (5) strategies to strengthen resilience.

**Conclusion:**

Students need a caring and supportive environment; interaction that is constructive, acknowledges human rights and makes use of appropriate non-verbal communication. The educator must display qualities such as love and care, respect, responsibility, morality, patience, being open to new ideas, motivation, willingness to ‘go the extra mile’ and punctuality. Students reported on various ways how they manage to stay resilient. It thus seems that basic elements required in an effective educator–student relationship to strengthen the resilience of students include the environment, interaction, educator and student’s qualities and resilience.

## Introduction and background

Effective educator–student relationships can have an impact on the resilience of students. Protective factors such as caring relationships, high expectations and opportunities to participate and contribute improve not only students’ academic performance but also strengthen their resilience (Hanson, Austin & Lee-Bayha 2004; Hurlington 2010). Caring educators who show concern for students and act as confidants, role models and mentors may thus contribute to students’ capacity to overcome personal vulnerabilities and environmental adversities (Wang, Haertel & Walberg 1994). Therefore, educators can have a positive effect not only on students’ academic achievement and behaviour but also on their resilience and long term success in life (Hanson & Austin 2003).

Three elements seem to be important in the educator–student relationship, namely: (1) the teaching–learning environment, (2) exchange of information and (3) mentor–peer association (Anderson & Carta-Falsa 2002). In addition, Beutel (2009) found 5 main categories in exploring educators’ understanding of their relationships with students, namely: (1) information providing, (2) instructing, (3) facilitating, (4) guided participation and (5) mentoring. Literature on the educator–student relationship furthermore includes resilience as an important factor to promote positive and supportive relationships between educators and students (Hurlington 2010; Johnson 2008). It seems that educators play a crucial role in building an environment that buffers students against adversity, and fosters the psychological well-being and healthy development they need in order to learn (Bunn 2000). Educators who form caring relationships with students and who create positive learning environment have a strong influence on students and on learning outcomes (Downey 2008; Sosa 2011).

McLaughlin and Talbert (1993) mention that educators need to create an environment that support students’ resilience by demonstrating high expectations and trust, promoting caring relationships among colleagues and providing ongoing opportunities for small groups to reflect and make decisions together. In addition, Black (1999) state that educators who listen, assess individuals’ strengths and create ways for students to express themselves and demonstrate their understanding, cultivate caring students and ensure that students engage and take more risks in classroom activities. Wade and Kasper (2006) also note that educators should promote trust, sharing and respect for an effective relationship with students. The above statements by different authors make it clear that the educator must possess certain qualities in order to ensure that an effective educator–student relationship exists. Educators need to listen to their students, engage them as fellow human beings, recognise and understand their perspectives and world views, and attend to their relational needs (Johnson 2008).

Research on this topic available at the onset of this study either explored the educator–student relationship between the clinical facilitator and students in the clinical environment (Wade & Kasper 2006) or explored the schooling years of children (Beutel 2009; Hughes 2012) and undergraduate or graduate students at universities (Anderson & Carta-Falsa 2002; Barta 2010; Chang & Davis 2009; Petrus *et al*. 2012; Redmond & Sorrell 1996; Rennie & Glass 2001). Del Prato *et al*. (2011) in turn explored the clinical environment of nursing students together with the faculty–student relationship.

It was evident that little has been explored about the relationship between the educator and student within a private nursing school with regard to the resilience of students. There seems to be a specific need for such research in South Africa. The current situation in nursing education in South Africa is that nursing students lack positive role models, experience high stress levels, and are not adequately prepared to fulfil their roles once qualified (South African Department of Health 2013). In addition, nurse educators face challenges such as having to cope with increasing student numbers. In this situation, private nursing schools play a major role in preparing large numbers of lower category nurses, such as auxiliary nurses, and are called upon to implement strategies, such as effective educator–student relationships, to not only contribute to the production of highly skilled nurses but also to contribute to the personal growth and resilience of these nurses (South African Department of Health 2013).

### Problem statement

In South Africa, as in other developing countries, limited research has been conducted in private nursing schools with regard to the educator–student relationship (Freemen, Anderman & Jensen 2007). An effective educator–student relationship is a key factor to ensure a positive learning climate where learning can take place (Freeman *et al*. 2007). A positive and supportive classroom environment improves students’ social and emotional well-being and ensures their motivation to continue trying; it builds trust in students’ abilities and also strengthens their ability to effectively deal with daily stress (Sosa 2011). Johnson (2008) emphasises the importance of a positive and supportive relationship between educators and students which will contribute to strengthening students’ resilience.

Although literature could be found on the basic elements required for an effective educator–student relationship (Anderson & Carta-Falsa 2002; Caballero 2011), very limited research has been conducted from a nursing students’ perspective, especially enrolled nursing auxiliary students in a private nursing school. As an educator, the researcher experienced that these students tend to depend on educators for guidance, support, care and understanding, necessitating a sound educator–student relationship. However, many educators tend to see students as merely the recipients of information and not as part of the educator–student relationship. Educators seemed to be in need of information to guide nursing students at private nursing schools in building effective educator–student relationships and to strengthen the resilience of students.

Furthermore, this research was conducted as a sub-study in the RISE project. The RISE project is concerned with strengthening the resilience of health caregivers and risk groups (Koen & Du Plessis 2011). According to Koen and Du Plessis (2011), the resilience of health caregivers needs to be strengthened in order to prevent threats to their well-being that may lead to lowered quality of healthcare. Therefore, strengthening students’ resilience from the beginning of their nursing career through a positive and supportive educator–student relationship can improve their well-being as well as uplift the quality of education, thereby improving the quality of patient care delivered.

It was thus clear that there was a need for research to explore nursing students’ view with regard to the basic elements required in an effective educator–student relationship to strengthen their resilience and to improve the educator–student relationship.

### Purpose of the research

The purpose of this research was to explore and describe nursing students’ view with regard to the basic elements required in an effective educator–student relationship to strengthen their resilience and the educator–student relationship. It was expected that this information would contribute to formulating recommendations for strengthening the resilience within the educator–student relationship and to improve the existing educator–student relationship.

### Definition of key concepts

Key concepts relating to this research are defined, namely, educator–student relationship, nursing education institute (NEI) and resilience.

Educator–student relationship: According to Gravett (2005), the educator is someone who is assigned the role of a leader or orchestrator of educational events for students. In this study, student refers to the enrolled nursing auxiliary student who is enrolled for a one-year nursing programme at a nursing school. Should this person also be registered as a learner with the South African Nursing Council (SANC)?

The Oxford Dictionary (2015) defines a relationship as ‘the way in which two or more people or things are connected, or the state of being connected’, and this can include the relationship between educator and student. In this study the relationship refers to the interpersonal and professional relationship between the educator and student.

The educator–student relationship is thus the academic relationship between an educator and a student. For the purpose of this research, an ‘effective’ educator–student relationship is evident when the student reaches academic outcomes, grows as a person and his or her resilience is strengthened.

NEI: In the context of this study, an NEI refers to an educational nursing institution of learning, which provides education and training for student nurses as regulated by the Nursing Act, No. 33 of 2005 (South Africa 2005). In this research, NEI particularly refers to the private nursing education institution in the North West Province.

Resilience: Resilience is defined as the ability to adapt well in the face of adversity, trauma, tragedy, threats or even significant sources of stress (De Chesnay 2005). It is the interaction between the person and the environment, and can be related to self-agency. Henderson and Milstein (1996) in turn broadly defines resilience as the capacity to spring back, rebound, successfully adapt in the face of adversity, and develop social, academic, and vocational competence despite exposure to severe stress or simply to the stress that is inherent in today’s world. In this study resilience refers to the ability of the educator and student to cope effectively with stressors.

### Contribution to the field

An exploration and description of what nursing students perceive as basic elements required for an effective educator–student relationship to strengthen their resilience enabled the formulation of recommendations to strengthen the resilience within the educator–student relationship and to improve the existing educator–student relationship.

### Research method and design

The research method and design are discussed by referring to the research design, population and sample, data collection, data analysis and the context of the study.

### Research design

This study followed an explorative, descriptive and contextual qualitative design.

The researcher selected this design to gain in-depth understanding of the phenomenon under investigation, namely, the basic elements in an effective educator–student relationship in the context of nursing education to strengthen the resilience of nursing students and to improve the educator–student relationship.

### Population and sample

The population used for this study comprised the enrolled nursing auxiliary students of a private nursing education institution (NEI) in a town in the semi-urban North West Province of South Africa. Purposive sampling was implemented. The sample consisted of an enrolled nursing auxiliary group of 40 students. The following inclusion criteria were used:

1. Participants had to be enrolled as auxiliary nursing students with the specific nursing school where the study was conducted.
2. Participants had to be enrolled in the auxiliary nursing programme for at least 3 months to ensure that rich information could be obtained.

### Data collection

The researchers applied the World Café as data collection method. The World Café has been developed as a brainstorming tool to generate ideas and comments about a specific topic (Brown, Homer & Isaacs 2005). According to Brown *et al*. (2005), the World Café method is a living network of conversations for leading collaborative dialogue, sharing knowledge and creating possibilities for action in groups of all sizes around questions that matter. Permission to use the World Café name, logo, method and materials was obtained from these authors.

Although the World Café was originally not developed as a research data collection method, the researchers saw the potential of this method to yield rich data essential to the educator–student relationship and decided to apply this method for the purpose of data collection. This method was appropriate to the study because a large quantity of rich data could be collected over a short period of time and generated ideas and comments of 40 enrolled nursing auxiliary students on specific topics.

In applying this method, the classroom environment was arranged according to the setup in a café. The tables were placed into five groups of eight participants with one poster, coloured markers and refreshments. Participants were asked to sit eight to a table and had a series of conversational rounds; lasting from 10 to 15 min each, with one discussion question at each table.

Based on the research objectives as well as guided by literature, the following discussion questions were used in the World Café discussions to explore enrolled auxiliary nursing students’ at a private NEI in the North West Province view on the basic elements of an effective educator–student relationship to strengthen resilience and improve the relationship:

What is needed in the teaching–learning environment to improve the relationship with your educator?

What type of interaction will improve your relationship with your educator?

What qualities must your educator display to improve the relationship between the educator and student?

How do you manage to stay resilient?

What suggestions can be implemented in the educator–student relationship to strengthen your resilience as students?

At the end of each round, one person remained at each table as the host, while the other seven travelled to the next table. Table hosts welcomed the next group of participants to their tables and shared the information of that table’s conversation so far. The newly arrived group then related to any of the written ideas and added new ones. This process continued until each group had been at all tables where the five questions were presented. These group discussions were followed by a class discussion session of 30–60 min where participants reflected on the whole process and explained, clarified and verified their findings and ideas written down on the posters. The discussion session was audio recorded and then transcribed. Field notes were taken throughout the process.

A trial run was conducted a month before the actual data collection commenced. According to Brink (2012), a trail run is a small-scale version of the major study conducted on a limited number of participants. The data collected during the trial run have not been included as part of the findings of the actual study as changes had to be made to the questions as initially formulated, namely, that it was revised to be more open-ended and clear in meaning.

### Data analysis

Data were analysed by using Creswell’s (2009) steps in content analysis. This involved moving deeper and deeper into understanding the data, representing the data and making interpretations of the larger meaning of the data. Inductive data analysis is used for qualitative research, which includes building patterns, themes and categories from the bottom-up and organising data into more abstract units (Creswell 2009:175). Data analysis was done by reading through each poster individually and the transcription of the group discussion, moving into a deeper understanding of what participants perceived to be basic elements of an effective educator–student relationship to strengthen resilience and to improve the relationship. The data set was interpreted as a whole, and not necessarily per question. However, participants’ views could be grouped together naturally in themes and categories that closely resemble the central theme of each question asked during the World Café workshop. The researcher made use of a co-analyst and both analysed data according to a data analysis work protocol. The researcher and co-analyst started to build patterns, themes and categories as evident from the data. After the researcher and co-analyst analysed the data independently, a meeting was scheduled to reach consensus on the themes and categories that emerged from the data collected.

### Context of the study

The study was conducted in a private nursing educational institute (NEI) in the North West Province, and this consequently forms the context of the study. The NEI offers the enrolled nursing auxiliary course for a period of 1 year. There are two intakes of 40 enrolled nursing auxiliary students per year. The staffing includes one principal, two tutors, two clinical tutors, one enrolled nursing auxiliary, one administrative assistant and one cleaner. The NEI is an accredited nursing education institution. The demographic profile of enrolled nursing auxiliaries is that they are mostly black females between the ages of 18–54 years, distributed from all over South Africa. Applicants must have at least a grade 10 certificate.

## Ethical considerations

The researcher obtained ethical clearance from the North-West University’s ethics committee before conducting the study to ensure that all ethical considerations have been adhered to. The researcher also obtained permission from the institution where the research was conducted.

### Potential benefits and hazards

Participants were informed that they might experience slight emotional discomfort due to their participation. A professional counsellor was on standby for debriefing and support. Ground rules to discuss confidentiality were discussed with the participants before the onset of data collection. Data were collected in a private, comfortable venue.

### Recruitment procedure

An information session was held with prospective participants to inform them about the study as well as to explain the concept of resilience before the commencement of data collection.

### Informed consent

The researcher obtained voluntary informed written consent from the prospective participants. Participants were given time to consider the invitation, and the researcher emphasised that they could withdraw or abstain at any time without discrimination or prejudice. No participant was manipulated or forced to participate in the study.

### Data protection

No personal details of participants were revealed in any reports, and the privacy of participants was respected throughout the study. The data are being stored on a password-protected computer and in a locked cupboard, and will be destroyed after a period of 7 years.

### Rigour

The researcher ensured that the study complied with the criteria for trustworthiness namely: truth value, applicability, consistency and neutrality (Lincoln & Guba 1985). In this study, the researcher ensured truth value by obtaining the experiences as it is lived and perceived by the participants, which reflects the credibility of the findings. According to Lincoln and Guba (1985), member checks is used as a technique to establish credibility. The researcher used a feedback and discussion session where participants had the opportunity to reflect on data collected which ensured that participants’ views were accurately recorded.

Transferability is reached if the study is one in which findings are relevant in other contexts (Lincoln & Guba 1985). A technique used to establish transferability is thick description. According to Thomas and Brubaker (2000), thick description allows the reader to enter the research context. With regard to transferability, the researcher presented the data sufficiently and descriptively to make it possible for another to make a comparison if needed.

Consistency checks included having an independent coder checking the category descriptions and text belonging in those categories. The researcher made use of a feedback session where the table host had the opportunity to report on ideas and comments written down on the posters, to verify and clarify the data collected.

Lincoln and Guba (1985) deemed that a confirmable study is objective. The researcher reached confirmability through ensuring that the findings, conclusions and recommendations were supported by data obtained and the link between the researcher’s interpretation and actual events. Findings could not be generalised, but the research process is discussed in detail so that the application of this research in a similar context will be possible. In order to further ensure trustworthiness, the researcher used multiple data sources to integrate the findings and existing literature.

## Results

**FIGURE 1:**
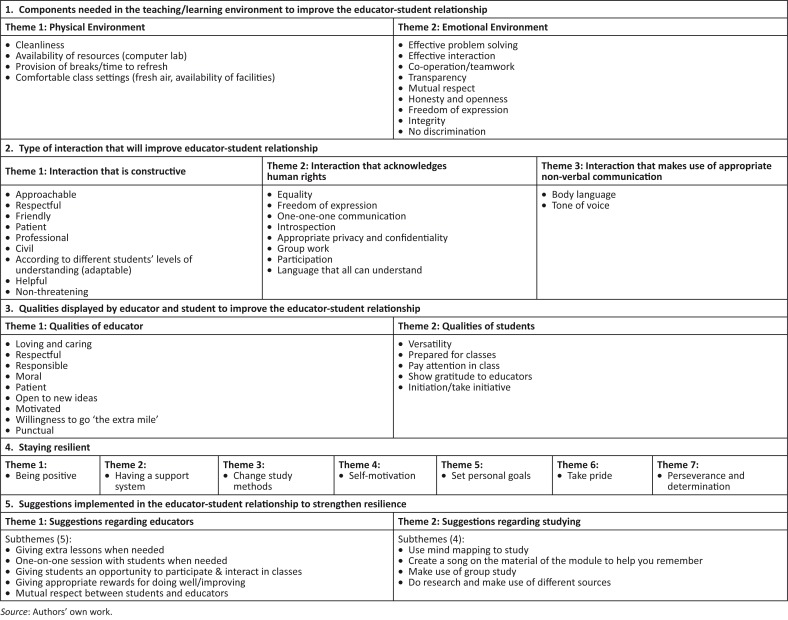
Themes: World café posters and discussions

Five main themes emerged from the data, which could be divided into categories (see Figure 1). These themes and categories are introduced and discussed, and relevant quotes from the World Café discussions are provided as evidence.

### Theme 1: Teaching–learning environment

Findings relating to the teaching–learning environment could be divided into two categories namely the physical environment and the emotional environment. Participants reported that the physical environment of the school needs to be neat and tidy. With regard to the availability of resources participants reported a strong need for a computer laboratory in the school to accommodate all students. Students want to have comfort breaks and fun through and between lessons. They want to play games to refresh their minds, body and souls even doing exercises before class. Participants stated that the class needs to have a comfortable environment with fresh air. The windows must be open, or the air-conditioning must be switched on. The rest rooms also need to be easy accessible for the students to prevent students from missing a lot of work while using the restrooms or going out to drink water. For them, such basic elements are important to improve the relationship between the educator and student. The participants mentioned, for example:

“The physical environment means the whole, the building, desks, chairs, lighting and the air-conditioner”. (Data collection method was a once-off group discussion)

Participants reported that the emotional environment included effective problem-solving, effective interaction, co-operation, transparency, mutual respect, honesty and openness, freedom of expression, integrity and no discrimination. Students feel that the more they are willing to interact the more they get to know each other. They believe that through being open towards their educator, the educator will be able to discover problems and thereby help them to solve it. Through effective interaction, the educator and student understand each other better, improving the educator–student relationship. Participants reported that:

“God helps those who want to help themselves. So you cannot expect your educator to help you if you also don’t make an effort”. (Once-off group discussion)

### Theme 2: Educator–student interaction

The educator–student interaction is divided into three main categories, namely, interaction that is constructive, interaction that acknowledges human rights and interaction that makes use of appropriate non-verbal communication.

Participants reported that they need interaction that is constructive and which included that the nurse educator is approachable, respectful, friendly, patient, professional, civil, adaptable, helpful and non-threatening. The educator needs to respect students in order for them to respect him or her. Students believe that the educator must be patient because their pace in learning differs. For example, as participant reported:

“When students encounter a problem the educator must not threaten them or make them feel small or insecure but in return help them to solve the problem”. (Once-off group discussion)

Participants furthermore mentioned a desire for interaction that acknowledges human rights such as equality, freedom of expression, one-on-one communication, introspection, privacy and confidentiality, group work, participation and language that all can understand. The following words of a participants illustrate this finding:

“When you have a problem, for the sake of students on different levels, I must not be afraid to talk to my educator, so that she can know me better and know my problems”. (Once-off group discussion)

and:

“Know who you are and what you want and what you want life to reap out of you. That means you must be able to go forward and know where you want to grow in although you don’t know where you are going to, but you know where you coming from”. (Once-off group discussion)

Interaction that makes use of appropriate non-verbal communication includes the use of appropriate body language and tone of voice. The educator must make use of appropriate body language which is non-discriminating and friendly, thereby creating an open and accessible learning environment. A participant shared the following:

“She must be friendly when she enters the door so that all of us can be relaxed”. (Once-off group discussion)

### Theme 3: Educator and student qualities

The category educator qualities refer to the qualities an educator needs to display to improve the relationship with students. During data analysis two categories emerged from data namely qualities of the educator and qualities of students.

Participants expected the educator to display qualities of love and care, respect, responsibility, morality, patience, open to new ideas, motivated, willing to “go the extra mile” and punctuality. They commented:

“Because if the educator comes in rude, we will assume that she is angry and we will not enjoy the class. The educator must be friendly and positive”. (Once-off group discussion)

Participants expected of themselves as students to demonstrate versatility, be prepared for classes, pay attention in class, show gratitude to educators and take initiative. A participant mentioned:

“The learner must come prepare to class, for instance, if the educator says please look at learning unit 1.2 and 3 and 4, just go through it”. (Once-off group discussion)

### Theme 4: Managing to stay resilient

Participants reported various ways how they manage to stay resilient. This included being positive. Participants mentioned that they need to be positive by acknowledging and accepting the situation they’re in including being adaptable, for example:

“Have a positive attitude and approach to life”. (Once-off group discussion)

Having a support system was another way to stay resilient. This refers to students seeking support or help from churches, clinics for counselling and social workers. Some participants also mentioned that they seek help from their educator. A participant mentioned:

“Seek help from a church like a pastor to help you cope with the situation”. (Once-off group discussion)

Changing study methods was also seen as a way to stay resilient. Participants who do not perform well said that they needed to change their study methods. For example, they either study in groups or have one-on-one sessions with their educator, as evident from the following quote:

“Form a group to study or have a one-on-one with your tutor to help you”. (Once-off group discussion)

Participants also said they manage to stay resilient by motivating themselves to be productive having a positive approach to life and finding ways to solve any misunderstandings or problems and always keeping their morale high. Participants said that they talk to people who experience similar problems and never allow anyone to make them feel inferior because they are special. A participant mentioned:

“Be able to uplift yourself”. (Once-off group discussion)

In addition, participants managed to stay resilient by setting personal goals for themselves. Participants also mentioned that they do not compare them with others because everyone is different in their own unique way. They believe in having faith and courage in themselves help them to stay resilient. The following quote illustrates this finding:

“If someone is performing well in their studies, asks that person how do they do that, how do they make it for achieving”. (Once-off group discussion)

Another way to stay resilient is being proud and taking pride in what students have achieved or what they are going to achieve at the end. They stated:

“Be proud and take pride”. (Once-off group discussion)

In using perseverance and determination, students think positively about themselves. A participant mentioned:

“You must have patience and determination in everything that comes your way because failure is not your destiny but success is your destiny and a way forward. Look at things from a positive perspective”. (Once-off group discussion)

### Theme 5: Strategies to strengthen resilience

In the last theme, participants reflected on strategies or suggestions nurse educators can use to help them strengthen their resilience as nursing students. Two categories emerged from the data. Participants reported strategies that educators can implement, namely giving extra lessons when needed, one-on-one sessions with students when needed, giving students an opportunity to participate and interact in classes, giving appropriate reward for doing well or improving and mutual respect between educators and students.

Participants also mentioned strategies students can implement, namely, using different study methods can improve their resiliency skills, using mind mapping to study, creating songs on the material of the module to help them remember, making use of group study, doing research and making use of different sources.

## Discussion

The results are discussed by providing an outline of the results and by referring to practical implications.

### Outline of the results

It is evident that certain basic elements need to be in place to ensure that the educator–student relationship remains effective and that the resilience of students is strengthened.

The first element in the relationship is the environment. This environment includes both the physical and emotional components in teaching–learning. Students need a caring and supportive environment including enough space, lighting and ventilation, as well as warmth, support, caring and trust. Literature confirms that the physical and emotional environment plays an important role in creating a safe and comfortable learning climate in which belonging, cooperation and appreciation for each other is emphasised and which contributes to a positive educator–student relationship (Brownlie & King 2000; Cooper 2004; Halarie & Cross 2012).

The second element is interaction between the educator and student. Students reported that they need interaction that is constructive, interaction that acknowledges human rights and interaction that makes use of appropriate non-verbal communication. Such interactional processes, also mentioned by Kumpher (1999) include role modelling, teaching, giving advice, empathetic and emotionally responsive caregiving, creating opportunities for meaningful involvement, effective supervision and disciplining, and reasonable developmental expectations. Del Prato *et al*. (2011) also explain that the educator contributes to an effective educator-student relationship through being approachable and respectful, through showing confidence in students, correcting students without being personal, listening to students, acknowledging where needed, showing a genuine interest in students and through being patient with students.

The third element of the relationship qualities refers to both the educator and student. Students mentioned that the educator must display qualities such as love and care, being respectful, responsible, moral, patient, and open to new ideas. In addition, students stated that their own qualities should include versatility, being prepared for classes, paying attention in class, showing gratitude to educators and initiative. Therefore, it seems that strengthening both the educator and students’ internal characteristics will improve the educator–student relationship and promote positive and successful academic outcomes. Similar views could be found in literature. Caring and supportive educators create qualitatively different classroom environments that feel warm, encourage students to behave in responsible ways and emphasise learning over performing (Davis 2009). The nurse educator who is most effective in creating an effective environment for learning to takes place is one who is respectful towards learners uniqueness and abilities, one who is usually wise, non-judgmental, generous, confident, honest, willing to take risks, willing to show forth without showing off and motivated to educate (Meyer & Van Niekerk 2008). Also, students who believe in their own effectiveness and who take initiative contribute to their own motivation, skills and success (Hupfeld 2010).

Resilience is also a crucial element in the educator–student relationship. Students reported on various ways how they manage to stay resilient, namely: being positive, having a support system, change study methods, self-motivation, set personal goals, take pride, and perseverance and determination. Similarly, Hupfeld (2010) stated that educators play a very important role in students’ lives by modelling resilience skills. By engaging in goal-oriented behaviours such as identifying goals, making plans and providing feedback, they provide models of these processes for students (Anderson & Carta-Falsa 2002; Hupfeld 2010).

### Practical implications

Through forming strong, caring and supportive relationships, educators contribute to students feeling safer and more secure in the educational setting, feeling more competent, making more positive connections and improving academic outcomes. Creating a caring and supportive environment where learning can take place not only improve the relationship but also have a positive effect on the students’ academic performance and resilience.

Educators that build effective relationships with students and create a positive learning climate establish an atmosphere characterised by mutual support, caring and understanding, all of which are foundational to a sound educator–student relationship. Resilient students who have positive attitudes believe that when they try, they will succeed. Academic success and resilience are fostered by the development of good study strategies and self-regulation of academic work.

Nurse educators should strengthen the resilience of nursing students by being a role model for them. To become excellent, caring and responsible nurses, students need to understand the importance of effective interpersonal relationships, something that will impact the rest of their nursing career.

### Limitations of the study

The researchers acknowledge that this research is limited to only one private nursing education institution and that the findings cannot be generalised. However, this article provides valuable information that can be considered by educators in semi-urban contexts in private nursing educations with regards to strengthening the educator–student relationship.

### Recommendations

Educators should be informed on how to establish a positive and effective educator–student relationship and what effects it has on students. Educators need to implement strategies to strengthen the resilience of our nursing students and improve their relationship with students. Nursing students need to be made aware of how to stay resilient and what strategies to follow to strengthen their resiliency.

## Conclusion

The purpose of this research, namely, to explore and describe nursing students’ view with regard to the basic elements required in an effective educator–student relationship to strengthen students’ resilience, has been reached. Further research needs to be done on how to measure the existing educator–student relationship existing in the classroom, also in relation to resilience.

## References

[CIT0001] Anderson L.E. & Carta-Falsa J, 2002, ‘Factors that make faculty and student relationships effective’, *College Teaching* 50(4), 134–138. 10.1080/87567550209595894

[CIT0002] Barta B.L.R, 2010, *‘Certified nurse educators: Espoused and enacted teacher beliefs and the role they play in understanding relationship with nursing students’*, PhD dissertation, The Ohio State University, Columbus, O.H.

[CIT0003] Beutel D, 2009, ‘Teachers’ understanding of their relationships with students: Pedagogic connectedness’, *The International Journal of Learning* 16(3), 507–518.

[CIT0004] Black S, 1999, ‘Teachers who connect with kids’, *American School Board Journal* 186(9), 42–44.

[CIT0005] Brink H, 2012, *Fundamentals of research methodology for health care professionals*, Juta, Cape Town.

[CIT0006] Brown J., Homer K. & Isaacs D, 2005, *The world café: Shaping our futures through conversations that matter*, Berrett-Koehler Publishers, San Francisco, California.

[CIT0007] Brownlie F. & King J, 2000, *Learning in safe schools: Creating classrooms where all students belong*, Pembroke Publishers, Markham.

[CIT0008] Bunn S, 2000, *Keeping kids connected. How schools and teachers can help all students feel good about school and why that matters*, Oregon Department of Education, Salem, Oregon.

[CIT0009] Caballero J.A.R, 2011, *‘The effects of the teacher-student relationship, teacher expectancy, and culturally-relevant pedagogy on student academic achievement’*, Doctoral dissertation, University of Redlands.

[CIT0010] Chang M. & Davis H, 2009, ‘Understanding the role of teacher appraisals in shaping the dynamics of their relationships with students: Deconstructing teachers’ judgments of disruptive behavior/students’, in Schutz P. & Zembylas M. (eds.), *Advances in teacher emotion research: The impact on teachers’ lives*, (pp. 95-127). Springer, New York 10.1007/978-4419-0564-2.6

[CIT0011] Cooper B, 2004, ‘Empathy, interaction and caring: Teachers’ roles in a constrained environment’, *Pastoral Care in Education* 22(3), 12–21. 10.1111/j.0264-3944.2004.00299.x

[CIT0012] Creswell J.W, 2009, *Research design. Qualitative, quantitative, and mixed methods approaches*, SAGE, London. 10.7748/nr.12.1.82.s2 28718745

[CIT0013] Davis H, 2009, ‘Caring teachers’, in Anderman E. & Anderman L. (eds.), *Psychology of classroom learning: An encyclopaedia (PCL)*, Volume 1 (pp. 138-141). Macmillan Reference, New York.

[CIT0014] De Chesnay M, 2005, *Caring for the vulnerable: Prospective in nursing theory, practice and research*, Jones and Barlett Publishers, London.

[CIT0015] Del Prato D., Bankert E., Grust P. & Joseph J, 2011, ‘Transforming nursing education: A review of stressors and strategies that support students’ professional socialization’, *Advances in Medical Education and Practice* 2, 109–116. 10.2147/AMEP.S18359 23745082 PMC3661250

[CIT0016] Downey J.A, 2008, ‘Recommendations for fostering educational resilience in the classroom’, *Preventing School Failure* 53(1), 57 10.3200/PSFL.53.1.56-64

[CIT0017] Freemen T.M., Anderman L.H. & Jensen J.M, 2007, ‘Sense of belonging in college freshmen at the classroom and campus levels’, *The Journal of Experimental Education* 75(3), 203–220. 10.3200/JEXE.75.3.203-220

[CIT0018] Gravett S, 2005, *Adult learning. Designing and implementing learning events. A dialogue approach*, Van Schaik, Pretoria.

[CIT0019] Halarie A. & Cross H.R, 2012, ‘Teaching and assessing in nursing: A worth-remembering educational experience’, *Health Science Journal* 1, 1–5.

[CIT0020] Hanson T.L. & Austin G.A, 2003, *Student health risks, resilience, and academic performance in California: Year 2 report, longitudinal analyses*, WestEd, San Francisco, C.A.

[CIT0021] Hanson T.L, Austin G. & Lee-Bayha J, 2004, *How are student health risks and resilience related to the academic progress of school*, WestEd, San Francisco, C.A.

[CIT0022] Henderson N. & Milstein M.M 1996, *Resilience in schools: Making it happen for students and educators*, Corwin Press, Thousand Oaks, C.A.

[CIT0023] Hughes J.N, 2012, ‘Teacher-student relationships and school adjustment: Progress and remaining challenges’, *Attachment & Human Development* 14(3), 319–327. 10.1080/14616734.2012.672288 22537527 PMC3340616

[CIT0024] Hupfeld K, 2010, *A review of the literature: Resilience skills and dropout prevention*. Scholar Centric, University of Colorado at Denver and Health Sciences Centre, Denver, Colorado.

[CIT0025] Hurlington K, 2010, ‘Bolstering resilience in students: Teachers as protective factors’, *The Literacy and Numeracy Secretariat* 25, 1–4.

[CIT0026] Johnson B, 2008, ‘Teacher-student relationships which promote resilience at school: A micro-level analysis of student’s views’, *British Journal of Guidance & Counselling* 36(4), 385–398. 10.1080/03069880802364528

[CIT0027] Koen M.P. & Du Plessis E, 2011, ‘Strengthening the resilience of health caregivers and risk groups’, Research proposal, North-West University, Potchefstroom Campus. Unpublished.

[CIT0028] Kumpher K.L, 1999, ‘Factors and processes contributing to resilience: The resilience framework’, in Glantz M.D. & Johnson J.L. (eds.), *Resilience and development: Positive life adaptations*, Kluwer Academic/Plenum Publishers, New York.

[CIT0029] Lincoln Y.S. & Guba E.A, 1985, *Naturalistic inquiry*, SAGE, Beverley Hills, C.A.

[CIT0030] McLaughlin M.W. & Talbert J.E, 1993, *Contexts that matter for teaching and learning*, Stanford University, Stanford, C.A.

[CIT0031] Meyer S. & Van Niekerk S, 2008, *Nurse Educator in practice*, Juta, Cape Town.

[CIT0032] Oxford Dictionary, 2015, viewed 21 October 2015, from http://www.oxforddictionaries.com/definition/english/relationship

[CIT0033] Petrus N.G., Su S., Chan V., Leung H. & Cheung W, 2012, ‘The development of perceived campus caring scale in a university-based sample in Hong Kong’, *Psychology & Psychotherapy* 2(1), 102.

[CIT0034] Redmond G.M. & Sorrell J.M, 1996, ‘Creating a caring learning environment’, *Nursing Forum* 31(4), 21–27. 10.1111/j.1744-6198.1996.tb00502.x 9052188

[CIT0035] Rennie L. & Glass N, 2001, ‘Effective communication and university progression: Reflections of mature aged women nursing students’, *Australian Electronic Journal of Nursing Education* 7(2), 23.

[CIT0036] Sosa T, 2011, ‘Students’ views on what identifies teachers as effective’, *Journal of Research in Education* 21(2), 118–132.

[CIT0037] South Africa, 2005, *Nursing Act 33*, Pretoria.

[CIT0038] South African Department of Health, 2013, *The national strategic plan for nurse education, training and practice 2012/3–2016/7*, viewed 21 October 2015, from http://www.sanc.co.za/archive/archive2013/NursingStrategy2013.html

[CIT0039] Thomas R.M. & Brubaker D.L, 2000, *Thesis and dissertations: A guide to planning, research and writing*, Bergin and Garbey, Wesport, Connecticut.

[CIT0040] Wade G.H. & Kasper N, 2006, ‘Nursing students’ perceptions of instructor caring: An instrument based on Watson’s theory of transpersonal caring’, *Journal of Nursing Education* 45(5), 162–168. 16722498 10.3928/01484834-20060501-05

[CIT0041] Wang M.C., Haertel G.D. & Walberg H.J, 1994, *Educational* *resilience in inner-city America: Challenges and prospects*, Erlbaum, Hillsdale, N.J.

